# Anthropometric Measures and Frailty Prediction in the Elderly: An Easy-to-Use Tool

**DOI:** 10.1155/2017/8703503

**Published:** 2017-11-20

**Authors:** Vera Elizabeth Closs, Patricia Klarmann Ziegelmann, João Henrique Ferreira Flores, Irenio Gomes, Carla Helena Augustin Schwanke

**Affiliations:** ^1^Graduate Program in Biomedical Gerontology, Institute of Geriatrics and Gerontology (IGG), Pontifical Catholic University of Rio Grande do Sul (PUCRS), Av. Ipiranga 6681, Prédio 81, 7 Andar, Sala 703, 90619-900 Porto Alegre, RS, Brazil; ^2^Postgraduate Program in Epidemiology and Postgraduate Program in Cardiovascular Sciences, Federal University of Rio Grande do Sul (UFRGS), Av. Bento Gonçalves 9500, Prédio 43-111, Agronomia, 91509-900 Porto Alegre, RS, Brazil; ^3^Department of Statistics, Federal University of Rio Grande do Sul (UFRGS), Av. Bento Gonçalves 9500, Prédio 43-111, Agronomia, 91509-900 Porto Alegre, RS, Brazil

## Abstract

**Purpose:**

Anthropometry is a useful tool for assessing some risk factors for frailty. Thus, the aim of this study was to verify the discriminatory performance of anthropometric measures in identifying frailty in the elderly and to create an easy-to-use tool.

**Methods:**

Cross-sectional study: a subset from the Multidimensional Study of the Elderly in the Family Health Strategy (EMI-SUS) evaluating 538 older adults. Individuals were classified using the Fried Phenotype criteria, and 26 anthropometric measures were obtained. The predictive ability of anthropometric measures in identifying frailty was identified through logistic regression and an artificial neural network. The accuracy of the final models was assessed with an ROC curve.

**Results:**

The final model comprised the following predictors: weight, waist circumference, bicipital skinfold, sagittal abdominal diameter, and age. The final neural network models presented a higher ROC curve of 0.78 (CI 95% 0.74–0.82) (*P* < 0.001) than the logistic regression model, with an ROC curve of 0.71 (CI 95% 0.66–0.77) (*P* < 0.001).

**Conclusion:**

The neural network model provides a reliable tool for identifying prefrailty/frailty in the elderly, with the advantage of being easy to apply in the primary health care. It may help to provide timely interventions to ameliorate the risk of adverse events.

## 1. Introduction

Frailty is common among the elderly, and several pathophysiological processes are related to its development. It was also observed that there is a close relationship between frailty and weight loss, sarcopenia, obesity, body composition, and nutritional aspects. Frail older people are more likely to become dependent and vulnerable to adverse health outcomes, such as disability, falling, the need for long-term care, and mortality [1]. However, thus far, health care systems do not fully consider this to be an important issue [2].

Although several tools to diagnose frailty have been developed [3], most of them, including the widely accepted Fried Phenotype, can be difficult to apply in clinical practice, especially in primary health care (PHC) settings [4].

In this context, anthropometry is a useful and easy-to-apply tool to assess nutritional status, functional decline, and chronic health conditions, which are important risk factors for frailty [5]. However, in specific scientific literature related to the area of geriatrics and gerontology, there is a lack of studies that provide in-depth information about the anthropometric parameters of frailty in the elderly [6], and the World Health Organization [7] emphasizes the need for values pertaining to specific populations, such as the elderly.

Faced with the difficulty of applying the Fried Phenotype in older adults who were assisted at PHC centers, the purpose of this study was to verify the discriminatory performance of anthropometric measures in identifying frailty in the elderly assisted at the Family Health Strategy [8] and to create an easy-to-use tool for this population.

## 2. Materials and Methods

### 2.1. Study Design and Population

This is a cross-sectional study that is part of the Multidimensional Study of the Elderly in the Family Health Strategy (EMI-SUS), whose methodology is described in Gomes et al. [9]. The Family Health Strategy is part of the Brazilian Unified Health System (Sistema Único de Saúde) [8]. The sample comprised 583 older adults aged 60 years or older (sample size calculated between 418 and 799). More details about health characteristics of individuals assessed are described in Closs et al. [10].

### 2.2. Data Collection Procedures

Sociodemographic data were obtained through a general questionnaire administered at the subjects' homes. A multidisciplinary team collected data for the determination of the Fried Phenotype [11] according to the following procedures: (A) self-reported unintentional weight loss in the past 12 months (weight loss); (B) grip strength (weakness); (C) self-reported exhaustion (exhaustion); (D) walking speed (slowness); (E) weekly energy expenditure (low physical activity). Frailty (outcome) was dichotomized as frailty (frailty + prefrailty ≥ 1 component) and robustness (0 components). Nutritionists, trained by the International Society for the Advancement of Kinanthropometry [12], collected 26 anthropometric measures: weight; height; knee height; circumferences of the arm, calf, forearm, hip, neck, thigh, and waist (at the umbilical level, at the smaller point, and at the midpoint between the iliac crest and the costal edge); abdominal, bicipital, calf, pectoral, suprailiac, subscapular, thigh, and triceps skinfolds; sagittal abdominal diameter at six points (at the umbilical level, at the smaller waist, at the midpoint between the iliac crest and the costal edge, at the iliac crest level, at the largest waist, and in the orthostatic position). The anthropometric measures used were chosen according to the International Standards for Anthropometric Assessment and considered all the basic measurements items, skinfolds, and circumferences most frequently used in the evaluation of adults and elderly [12].

### 2.3. Statistical Analysis

Data were presented as mean (standard deviation) or frequency (percentage). To build a predictive rule to detect frailty, we used both logistic regression (LR) and artificial neural network (NN). NN can generate models with better results since it can include interactions among predictors [13–15]. Predictive models should be tested in order to evaluate their prognostic ability. Therefore, the original sample (*N* = 583) was divided into a learning sample (*n* = 439) and a testing sample (*n* = 144). The common rule of four participants in the learning group to one participant in the test group was used and the samples were selected, divided by the frailty subgroups. That is, approximately 80% of the individuals classified as frail and 80% of those classified as robust were randomly selected from the original sample to form the learning sample. The learning sample was used to develop the tool to identify frailty and the test sample was used to compare the prognostic ability of the tool. LR models (unadjusted and adjusted for age and gender) were constructed for each anthropometric predictor (considered to be continuous) to evaluate their predictive ability with regard to frailty. The LR linear assumption was checked by incorporating a quadratic effect in the model and testing its significance (*P* < 0.05). In addition, the accuracy of each univariate model in correctly identifying older adults with frailty was assessed by estimating the area under the Receiver Operating Characteristic (_au_ROC) curve. ROC curves were generated using the predicted probabilities estimated by the LR models. Potential predictors to build a multivariable model were selected based on the significance level and clinical practice. First, predictors with *P* > 0.20 in the univariable model were discarded. Second, for the measurements that were taken at more than one distinct anatomical point, just the one most cited in the literature (including international guidelines) was selected.

The multivariable model was built using a backward strategy and keeping age and gender as potential confounders. Neural networks are generally formed by a three-layer neuron structure, and a similar network structure was used in this study. The Multilayer Perceptron (MLP) NN is formed of neurons in the input, hidden, and output layers. A feedforward NN with the backpropagation learning algorithm was used [13]. The final model was selected after a series of tests with different configurations over the hidden layer. The accuracy of the final models was assessed by _au_ROC curve, sensitivity (Se), specificity (Sp), and predictive values (positive and negative) [16]. _au_ROC > 0.7 was considered to indicate sufficient predictive accuracy [16]. Predictive rules were constructed based on the multivariable LR model and the NN model using three criteria: higher Youden Index (YiC), Se of at least 0.80 (SeC), and Sp of at least 0.60 (SpC) [17]. All data analysis was performed using SPSS for Windows 17.0 [18] and R 3.1.1 [19].

### 2.4. Ethical Considerations

The study was approved by the Research Ethics Committee of PUCRS (protocol number CEP-10/04967) and by the Municipal Health Secretary of Porto Alegre (protocol number 001.021434.10.7). Informed consent was obtained from all participants.

## 3. Results

A total of 439 older adults (learning sample) (63.8% female) with a mean age of 68.7 ± 7.2 years (ranging from ages 60 to 103) were included in the study. The estimated prevalence of frailty (frailty + prefrailty) among the elderly was 70.8% (95% CI: 66.3–75.1).  Table 1 presents the characteristics of the participants according to the frailty diagnosis.

Table 2 presents the results for both unadjusted and age-adjusted LR univariable models and the _au_ROC. The sample size varied for each predictor due to missing values. Those results show the ability of each anthropometric variable as predictor of prefrailty + frailty.

All anthropometric measures individually lacked adequate predictive accuracy (_au_ROC > 0.7). Therefore, a multivariable model was adjusted. Three predictors (neck circumference, suprailiac skinfold, and abdominal skinfold) were discarded because they present *P* > 0.20. The three waist circumference measurements generated *P* < 0.20. For the final model the midpoint between the iliac crest and the costal edge was chosen, due to its scientific relevance (most commonly cited in the literature). Likewise, the six sagittal abdominal diameter measurements had *P* < 0.20, and the selected one was at the umbilical level. Therefore, 16 predictors (weight, height, and knee height; arm, forearm, waist midpoint, hip, thigh, and calf circumferences; subscapular, pectoral, triceps, bicipital, thigh, and calf skinfolds; sagittal abdominal diameter at umbilical level and age) were included in the multivariable model (Table 2).

The multivariable model started with two components (linear and quadratic) for those predictors with the quadratic element in the univariable model and one component (linear) for the others. In the backward procedure, both the linear and the quadratic components could be removed from the model independently. The final model comprised weight (WE), waist circumference at the midpoint between the iliac crest and the costal edge (WC), bicipital skinfold (BS), sagittal abdominal diameter at the umbilical level (SAD), and age.

Table 3 individually presents the prognostic predictive ability for these five predictors and that of the final RL multivariable model (learning and test sample). The results are presented by each of the three criteria, namely, SeC, SpC, and YiC. It can be seen that the predictors alone did not achieve simultaneously satisfactory values of Se and Sp.

The estimated frailty probability produced by the final multivariable LR model can be obtained by the equation: predicted probability = exp(*g*)/(1 + exp(*g*)), where *g* = 15.639022 + 0.073423 *∗* BS  +  0.003939 *∗* SAD^2^ + 0.001822 *∗* WC^2^ – 0.350425*∗*WC – 0.0621702*∗*WE + 0.044818*∗*Age (years).

In the univariate models, the frailty predictive ability of the predictors achieved values that did not exceed 80.2% of Se, and Sp ranged from 31.3% to 75.0%. The final multivariable LR model (predictors grouped) is translated into an improved capability to predict frailty: approximately the same Se (80%) but with higher Sp (range 48–79.2%).

As shown at the bottom of Table 3, the test sample results for SeC were slightly higher in both Se and Sp (81.7% versus 48.5%) than those obtained in the learning sample (80.1% versus 48.0%), suggesting that these values could be used for classification. The final multivariable LR model presented an _au_ROC of 0.71 (95% CI 0.66–0.77).

**Figure 1:**
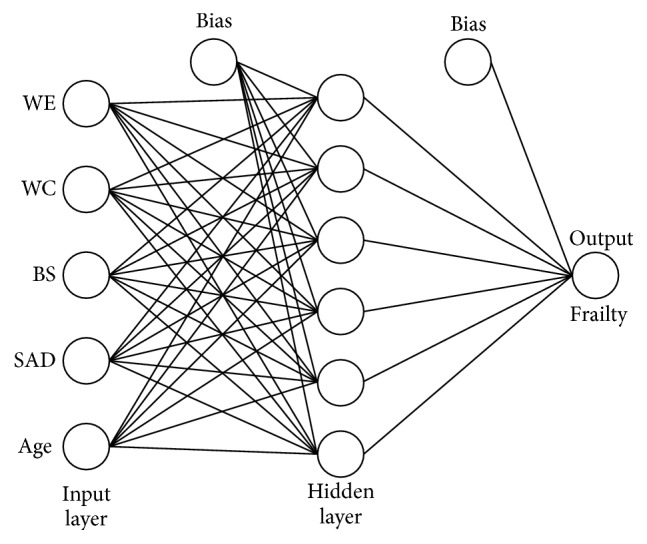
Neural network configuration of anthropometric measures and frailty in older adults who were assisted at primary health care centers. WE: weight; WC: waist circumference; BS: bicipital skinfold; SAD: sagittal abdominal diameter.

In order to further explore the predictive ability of the anthropometric predictors the five predictors left at the final multivariable LR model were used as the input layer of NN models that were built using an input layer, a hidden layer, and a single, continuous, output layer. We ran MLP models using three to six hidden layers. These limits were selected based on the amount of data available.  Figure 1 presents the architecture of the models.

The result from the final NN (output neuron) model is a continuous value ranging from zero (robust) to one (frail) depending on the predictors values. Although those values are not estimated as probabilities they have similar interpretation as the probabilities estimated by the LR model. The resulting equation from the NN final model is implemented in an Excel spreadsheet (Supplementary Appendix S1 in Supplementary Material available online at https://doi.org/10.1155/2017/8703503). The prognostic ability to predict frailty using the NN final model results is shown in Table 4 for the learning and the test samples. It can be seen that, in general, the results bare superiority to those found by the LR final model. In the learning sample, when the SpC cut-off point is used, the Se is higher for the NN model (Se = 0.797) when compared to the LR model (Se = 0.720). Also, for the SeC cut-off the NN model has Sp = 0.486 while the LR model has Sp = 0.480. In the sample test, all cut-off points resulted in better Se and Sp. The NN final model presented an _au_ROC of 0.78 (95% CI 0.74–0.82), indicating greater predictive accuracy (*P* < 0.001) than the LR model.

## 4. Discussion

The purpose of this study was to investigate the ability of anthropometric measures to predict prefrailty/frailty in older adults assisted at PHC centers. The main finding was that, among the 26 anthropometric measures analyzed, those that together predicted frailty were WE, WC, BS, and SAD for both the LR and the NN model. With regard to the method used to determine the predictive value of frailty, the NN proved to be more efficient than the LR model in predicting frailty in the older adults assessed.

To the best of our knowledge, this is the first investigation carried out in a sample of older adults who were assisted at PHC centers to verify the performance of several anthropometric measures in identifying frailty from the NN model. In addition, the resulting algorithm can be applied in the evaluation of older adults from the Family Health Strategy with satisfactory results.

Surveys often investigate the relationship of frailty with measures commonly used to evaluate nutritional status, such as the body mass index or body composition [20–22]. In our study, we used pure and simple measures, not combined into equations, with the objective of obtaining an algorithm that was as simple as possible, using easily obtained measurements in the PHC. The importance of identifying prefrailty or frailty in older adults is associated with the fact that interventions can help to prevent, delay, reverse, or reduce the severity of frailty as well as preventing or reducing adverse health outcomes in those whose frailty is not reversible. Effective interventions can promote benefits for elderly individuals, their families, and the whole society [23–25].

Anthropometric measures have been studied due to their ability to identify certain health parameters, such as nutritional status, due to their relationship with diseases and with physical function status [5, 26] and all conditions involved in the course of frailty [1]. However, the molecular, physiological, and clinical course of frailty, in turn, presents nonuniform features of pathways for an individual to become frail: people with the same level of frailty may have reached this stage in a variety of ways and present decline in their physiological reserve, whether in the neuromuscular, metabolic, or immune system [1]. Concerning the relationship between frailty and anthropometric measures, we observed that frailty stems from a set of observations and that the relationship between one such observation with certain anthropometric parameters is sometimes already known, while for others it is completely unknown [1]. This set of predictors that are connected to various shapes and weights motivated the use of NN, a model capable of handling nonlinear relationships [27].

Even considering that the strength or the predictive value is established by the set of predictors, it may be interesting to analyze the individual characteristics of the measures included in the final model (WE, WC, BS, and SAD).

It is known that WE on average tends to decrease after the age of 60 years, and the contribution of fat mass to this weight loss is relatively small, but the fat tends to be redistributed toward the abdomen, that is, increasing visceral fat deposits [5]. SAD and WC are measures that are positively related to abdominal adiposity [28], and some evidence has pointed to a possible connection between abdominal adiposity, cardiovascular diseases, and frailty in older people [22, 29, 30]. However, in aging, lean body mass is lost, while fat mass may be preserved or even increased. This state is named sarcopenic obesity [31] and it is known that sarcopenia is involved in the pathogenesis of frailty [1]. Previous study, evaluating the association of anthropometric measurements with frailty, in the same population, demonstrated that frailty was associated with muscle mass loss. The frail elderly had lower measures of size and complexion [32]. This pattern of increase of intramuscular and visceral fat with aging is accompanied by changes in subcutaneous fat, which can be evaluated through the measurement of BS [33]. Many frail elderly are thin, weak, and undernourished; however, there is also strong evidence that excessive adiposity contributes to frailty [21].

When grouped, it was possible to observe that the prognostic predictive ability of the predictors achieved better performance. The result of the LR was translated into improved predictive capability for prefrailty/frailty, with similar Se but higher Sp. However, it was noted that in relation to the LR model NN showed an improved predictive capability for prefrailty/frailty. For the three cut-off points assessed, both Se and Sp were considered superior and satisfactory to avoid false negative and positive screenings [17]. The accuracy of the final NN models, as assessed by _au_ROC, was higher than that of the LR model, overcoming the value considered to have sufficient predictive accuracy [16].

The main results of this paper were the demonstration of the following: (a) the performance capabilities of anthropometric measures to identify prefrailty/frailty; (b) the superior performance of the NN for prefrailty/frailty prediction with heterogeneous data—NN explores other relationships that can generate models with better results [15]; (c) early stages of frailty that are more commonly seen in the older adults of the community—therefore, identification and screening tools must be applied in this population [23].

This study has some positive aspects that must be noted. First, it includes the evaluation of a large set of anthropometric measurements. Second, the method used to investigate the ability of various anthropometric measures to identify prefrailty/frailty in the older adults is particularly innovative, specifically, the analytical methodology chosen to investigate this ability. Third, the model is capable of providing a noninvasive tool for the large-scale screening of prefrailty/frailty.

The limitations of the study include the following: (a) the cross-sectional design prevented the establishment of a cause-and-effect relationship; (b) the available data (small sample) restricted the configurations of the NN models. To overcome the second issue, we used the same variables of the LR model in the input layer and the backpropagation learning algorithm, method recommended for those cases [15]; however, further research should be carried out to resolve these limitations.

In conclusion, our data suggest that grouped anthropometric measures can be recommended as good predictors of prefrailty/frailty in older adults who were assisted at PHC centers. The possibility of having a simple tool in PHC to identify prefrailty/frailty allows for the implementation of an individual treatment plan that can prevent, reverse, or treat the complications that can lead to frailty while preserving the autonomy of the elderly.

In addition to promoting health benefits and quality of life in the elderly through the identification, prevention, and treatment of frailty, these measures can reduce health care costs. Further research is needed to establish the clinical utility of the instrument developed.

## Supplementary Material

EXCEL spreadsheet based on the parameters of neural network model and built to predict the outcome.

## Figures and Tables

**Table 1:** Sociodemographic characteristics of older adults who were assisted at primary health care centers.

Characteristics^*∗*^	Total *N* (%)	Frail + prefrail *N* = 311 *N* (%)	Robust *N* = 128 *N* (%)
Gender (female)	280 (63.8)	216 (69.5)	64 (50.0)
Age in years (mean ± SD)	68.7 ± 7.2	69.4 ± 7.7	66.7 ± 5.3
Age group (years)			
60–64.9	158 (36.0)	105 (33.8)	53 (41.4)
65–69.9	109 (24.8)	70 (22.5)	39 (30.5)
70–74.9	82 (18.7)	59 (19.0)	23 (18.0)
75–79.9	52 (11.8)	42 (13.5)	10 (7.8)
≥80	38 (8.7)	35 (11.2)	3 (2.3)
Race/ethnicity			
White	290 (67.3)	191 (62.6)	99 (78.6)
Black	76 (17.6)	44 (14.4)	10 (7.9)
“Mulatto”/brown-skinned	54 (12.5)	60 (19.7)	16 (12.7)
Native Indian	11 (2.6)	10 (3.3)	1 (0.8)
Education			
Illiterate	72 (16.7)	65 (21.3)	7 (5.5)
Incomplete elementary	117 (27.1)	88 (28.9)	29 (22.8)
Complete elementary	180 (41.7)	114 (37.4)	66 (52.0)
Complete middle school	38 (8.8)	23 (7.5)	15 (11.8)
Complete high school	21 (4.9)	13 (4.3)	8 (6.3)
Higher education	4 (0.9)	2 (0.7)	2 (1.6)
Marital status			
Married	162 (37.3)	107 (34.7)	55 (43.7)
Separated/divorced	71 (16.4)	45 (14.6)	26 (20.6)
Single	71 (16.4)	52 (16.9)	19 (15.1)
Widowed	130 (30.0)	104 (33.8)	26 (20.6)
Monthly income (MS^†^)			
Up to 2	382 (93.4)	273 (93.5)	109 (93.2)
>2 MS to 4	20 (4.9)	15 (5.1)	5 (4.3)
>4 MS to 6	7 (1.7)	4 (1.4)	3 (2.6)

*Notes*. ^*∗*^The number of subjects with missing values was eight for race/ethnicity, seven for education, five for marital status, and 30 for monthly income. ^†^MS: minimum salary = R$ 540 (=US$270).

**Table 2:** Logistic regression univariable models and _au_ROC results for the learning sample.

Anthropometric measures	*N*	Unadjusted		Adjusted for age	
		*β *(*P*)^*∗*^	_au_ROC (95% CI)	*β *(*P*)^*∗*^	_au_ROC (95% CI)
Weight^2^	436	0.001 (0.003)	0.58 (0.52–0.63)^‡^	0.001 (0.005)	0.63 (0.58–0.69)^†^
Height	436	−5.313 (<0.001)	0.64 (0.58–0.69)^†^	–4.731 (<0.001)	0.66 (0.61–0.72)^†^
Knee height	436	–0.098 (0.004)	0.60 (0.54–0.65)^‡^	–0.091 (0.008)	0.64 (0.58–0.69)^†^
Circumference					
Neck	429	–0.050 (0.128)	0.54 (0.48–0.60)	–0.032 (0.331)	0.60 (0.54–0.65)^‡^
Arm^2^	438	0.021 (<0.001)	0.61 (0.55–0.67)^†^	0.020 (0.001)	0.66 (0.60–0.71)^†^
Forearm	432	–0.097 (0.014)	0.58 (0.52–0.64)^‡^	–0.068 (0.100)	0.61 (0.55–0.66)^†^
Umbilical level^2^	417	0.002 (0.003)	0.58 (0.52–0.64)^‡^	0.002 (0.003)	0.65 (0.59–0.70)^†^
Smaller waist^2^	415	0.002 (0.011)	0.57 (0.52–0.63)^‡^	0.002 (0.009)	0.64 (0.58–0.69)^†^
Waist midpoint^2^	413	0.002 (0.007)	0.58 (0.53–0.64)^‡^	0.002 (0.005)	0.64 (0.58–0.70)^†^
Hip^2^	412	0.002 (0.009)	0.56 (0.50–0.62)	0.002 (0.010)	0.63 (0.58–0.69)^†^
Thigh^2^	416	0.006 (0.018)	0.57 (0.51–0.62)^‡^	0.005 (0.034)	0.62 (0.56–0.67)^†^
Calf^2^	434	0.015 (0.016)	0.55 (0.49–0.60)	0.014 (0.033)	0.61 (0.56–0.67)^†^
Skinfold					
Subscapular^2^	438	0.003 (0.031)	0.53 (0.48–0.59)	0.003 (0.045)	0.62 (0.56–0.67)^†^
Pectoral^2^	430	0.004 (0.085)	0.56 (0.50–0.61)	0.004 (0.091)	0.62 (0.57–0.68)^†^
Triceps	435	0.039 (0.005)	0.58 (0.53–0.64)^‡^	0.043 (0.003)	0.64 (0.59–0.70)^†^
Bicipital	432	0.059 (0.005)	0.58 (0.52–0.63)^‡^	0.067 (0.002)	0.65 (0.59–0.70)^†^
Suprailiac	417	0.008 (0.515)	0.53 (0.47–0.59)	0.014 (0.270)	0.60 (0.54–0.66)^‡^
Abdominal	418	0.001 (0.941)	0.52 (0.46–0.58)	0.008 (0.542)	0.59 (0.53–0.65)^‡^
Thigh	417	0.022 (0.033)	0.57 (0.51–0.62)^‡^	0.021 (0.042)	0.61 (0.56–0.67)^†^
Calf	428	0.048 (0.001)	0.60 (0.54–0.66)^‡^	0.045 (0.002)	0.64 (0.59–0.70)^†^
Sagittal abdominal diameter					
Umbilical level^2^	397	0.021 (0.012)	0.57 (0.51–0.63)^‡^	0.022 (0.010)	0.63 (0.57–0.69)^†^
Smaller waist^2^	397	0.026 (0.008)	0.56 (0.50–0.61)	0.027 (0.007)	0.62 (0.57–0.58)^†^
Midpoint^2^	397	0.021 (0.014)	0.57 (0.51–0.63)^‡^	0.022 (0.011)	0.63 (0.57–0.69)^†^
Iliac crest level^2^	397	0.022 (0.009)	0.58 (0.52–0.64)^‡^	0.023 (0.007)	0.63 (0.57–0.69)^†^
Orthostatic position^2^	400	0.013 (0.019)	0.58 (0.52–0.64)^‡^	0.014 (0.018)	0.63 (0.57–0.69)^†^
Larger waist^2^	397	0.023 (0.008)	0.59 (0.53–0.65)^‡^	0.023 (0.007)	0.64 (0.58–0.69)^†^

*Notes*. Exponent 2 means that the quadratic term of the predictor was included in the model, along with the linear term. ^*∗*^Logistic regression; ^†^*P* (_au_ROC) < 0.001; ^‡^*P* (_au_ROC) < 0.005.

**Table 3:** Prognostic ability of anthropometric measures as predictors of prefrailty + frailty in older adults who were assisted at primary health care centers.

Predicted probability of anthropometric measures	Frailty			Se	Sp	PPV	NPV
	Total prevalence *N* (%)	Yes *N* (%)	No *N* (%)				
Weight							
>0.6253^SeC^	335 (76.8)	247 (80.2)	88 (68.8)	0.802	0.313	0.737	0.396
>0.6777^SpC^	233 (53.6)	183 (59.4)	50 (39.4)	0.594	0.602	0.785	0.381
>0.7102^YiC^	188 (43.1)	156 (50.6)	32 (25.0)	0.506	0.750	0.829	0.387
Waist circumference at midpoint^ 2^							
>0.6161^SeC^	303 (73.4)	229 (80.1)	74 (58.3)	0.801	0.417	0.755	0.481
>0.6508^YiC^	254 (61.5)	197 (68.9)	57 (44.9)	0.689	0.551	0.775	0.440
>0.6680^SpC^	223 (54.0)	172 (60.1)	51 (40.2)	0.601	0.600	0.771	0.400
Bicipital skinfold							
>0.6211^SeC^	274 (71.5)	210 (77.5)	64 (57.1)	0.800	0.375	0.766	0.440
>0.6888^SpC^	239 (55.3)	188 (61.8)	51 (39.8)	0.618	0.602	0.786	0.399
>0.7138^YiC^	199 (46.1)	162 (53.3)	37 (28.9)	0.533	0.711	0.814	0.390
Sagittal abdominal diameter at umbilical level^2^							
>0.6095^SeC^	294 (74.2)	216 (73.5)	78 (61.9)	0.801	0.381	0.734	0.470
>0.6161^YiC^	284 (71.5)	213 (78.6)	71 (56.3)	0.786	0.437	0.750	0.486
>0.6637^SpC^	207 (52.1)	157 (57.9)	50 (39.7)	0.579	0.603	0.758	0.400
Logistic regression model (learning sample)							
>0.6158^SeC^	282 (71.2)	217 (80.1)	65 (52.0)	0.801	0.480	0.769	0.526
>0.6486^SpC^	245 (61.9)	195 (72.0)	50 (40.0)	0.720	0.600	0.795	0.496
>0.7137^YiC^	182 (46.0)	156 (57.6)	26 (20.8)	0.576	0.792	0.857	0.462
Logistic regression model (testing sample)							
>0.6158^SeC^	75 (72.1)	58 (81.7)	17 (51.5)	0.817	0.485	0.773	0.551
>0.6486^SpC^	66 (63.5)	51 (71.8)	15 (45.5)	0.718	0.546	0.772	0.473
>0.717^YiC^	49 (47.1)	38 (53.5)	11 (33.3)	0.535	0.667	0.775	0.400

*Notes*. Se: sensitivity; Sp: specificity; PPV: positive predictive value; NPV: negative predictive value; SeC: sensitivity ≈ 80%; SpC: specificity ≈ 60%; YiC: Youden Index. Exponent 2 means that the quadratic term of the predictor was included in the model, along with the linear term.

**Table 4:** Prognostic ability of anthropometric measures as predictors of frailty in older adults who were assisted at primary health care centers via artificial neural models.

Predicted probability of anthropometric measures	Frailty			Se	Sp	PPV	NPV
	Total prevalence *N* (%)	Yes *N* (%)	No *N* (%)				
Neural network (learning sample)							
>0.5648^SpC^	268 (67.2)	220 (79.7)	48 (17.9)	0.797	0.610	0.820	0.572
>0.7176^YiC^	217 (54.4)	188 (68.1)	29 (23.6)	0.681	0.764	0.866	0.516
>0.5621^SeC^	271 (68.3)	221 (80.1)	50 (41.3)	0.801	0.587	0.815	0.563
Neural network (testing sample)							
>0.5648^SpC^	69 (68.3)	54 (81.8)	15 (42.9)	0.818	0.571	0.782	0.625
>0.7176^YiC^	53 (53.0)	46 (69.7)	7 (20.6)	0.697	0.794	0.867	0.574
>0.5621^SeC^	72 (71.3)	54 (81.8)	18 (51.4)	0.818	0.486	0.750	0.586

*Notes*. Se: sensitivity; Sp: specificity; PPV: positive predictive value; NPV: negative predictive value; SeC: sensitivity ≈ 80%; SpC: specificity ≈ 60%. YiC: Youden Index.

## References

[1] Walston J., Hadley E. C., Ferrucci L. (2006). Research agenda for frailty in older adults: toward a better understanding of physiology and etiology: summary from the American geriatrics society/national institute on aging research conference on frailty in older adults. *Journal of the American Geriatrics Society*.

[2] Vellas B., Cestac P., Morley J. E. (2012). Editorial implementing frailty into clinical practice: we cannot wait. *The Journal of Nutrition, Health & Aging*.

[3] Kuzuya M. (2012). Process of physical disability among older adults-contribution of frailty in the super-aged society. *Nagoya Journal of Medical Science*.

[4] Abellan Van Kan G., Rolland Y., Bergman H., Morley J. E., Kritchevsky S. B., Vellas B. (2008). The I.A.N.A. task force on frailty assessment of older people in clinical practice. *The Journal of Nutrition, Health & Aging*.

[5] Seidell J. C., Visscher T. L. S. (2000). Body weight and weight change and their health implications for the elderly. *European Journal of Clinical Nutrition*.

[6] Barbosa A. R., Souza J. M. P., Lebrão M. L., Laurenti R., Marucci M. D. F. N. (2005). Anthropometry of elderly residents in the city of São Paulo, Brazil. *Cadernos de saúde públic*.

[7] World Health Organization (1995). Physical status: the use and interpretation of anthropometry. *Report of a WHO Expert Committee*.

[8] Macinko J., Harris M. J. (2015). Brazil's family health strategy - Delivering community-based primary care in a universal health system. *The New England Journal of Medicine*.

[9] Gomes I., Nogueira E. L., Engroff P., etal. (2013). The multidimensional study of the elderly in the family health strategy in Porto Alegre (EMI-SUS). *Pan American Journal of Aging Research*.

[10] Closs V. E., Ziegelmann P. K., Gomes I., Schwanke C. H. A. (2016). Frailty and geriatric syndromes in elderly assisted in primary health care. *Acta Scientiarum - Health Sciences*.

[11] Fried L. P., Tangen C. M., Walston J. (2001). Frailty in older adults: evidence for a phenotype. *The Journals of Gerontology: Medical Sciences*.

[12] Marfell-Jones M., Olds T., Stewart A., Lindsay Carter J. E. (2006). *International Standards for Anthropometric Assessment*.

[13] Puddu P. E., Menotti A. (2009). Artificial neural network versus multiple logistic function to predict 25-year coronary heart disease mortality in the Seven countries study. *European Journal of Cardiovascular Prevention and Rehabilitation*.

[14] Kupusinac A., Stokić E., Doroslovački R. (2014). Predicting body fat percentage based on gender, age and BMI by using artificial neural networks. *Computer Methods and Programs in Biomedicine*.

[15] Haykin S. (2001). *Redes Neurais: Princípios e Prática*.

[16] Wians F. H. (2009). Clinical laboratory tests: which, why, and what do the results mean?. *LabMedicine*.

[17] Blake H., McKinney M., Treece K., Lee E., Lincoln N. B. (2002). An evaluation of screening measures for cognitive impairment after stroke. *Age and Ageing*.

[18] SPSS Inc. *SPSS Statistics for Windows*, Version 17.0, Chicago, Illinois, USA, 2008.

[19] R Development Core Team (2011). *R: A Language and Environment for Statistical Computing*.

[20] Song X., Mitnitski A., MacKnight C., Rockwood K. (2004). Assessment of individual risk of death using self-report data: an artificial neural network compared with a frailty index. *Journal of the American Geriatrics Society*.

[21] Izawa S., Enoki H., Hirakawa Y. (2010). The longitudinal change in anthropometric measurements and the association with physical function decline in Japanese community-dwelling frail elderly. *British Journal of Nutrition*.

[22] Porter Starr K. N., McDonald S. R., Bales C. W. (2014). Obesity and physical frailty in older adults: a scoping review of lifestyle intervention trials. *Journal of the American Medical Directors Association*.

[23] Hubbard R. E., Lang I. A., Llewellyn D. J., Rockwood K. (2010). Frailty, body mass index, and abdominal obesity in older people. *The Journals of Gerontology. Series A, Biological Sciences and Medical Sciences*.

[24] Gill T. M., Gahbauer E. A., Allore H. G., Han L. (2006). Transitions between frailty states among community-living older persons. *JAMA Internal Medicine*.

[25] Chen X., Mao G., Leng S. X. (2014). Frailty syndrome: an overview. *Clinical Interventions in Aging*.

[26] Daniels R., Metzelthin S., van Rossum E., de Witte L., van den Heuvel W. (2010). Interventions to prevent disability in frail community-dwelling older persons: an overview. *European Journal of Ageing*.

[27] Landi F., Onder G., Russo A. (2014). Calf circumference, frailty and physical performance among older adults living in the community. *Clinical Nutrition*.

[28] Goulet E. D. B., Hassaine A., Dionne I. J. (2009). Frailty in the elderly is associated with insulin resistance of glucose metabolism in the postabsorptive state only in the presence of increased abdominal fat. *Experimental Gerontology*.

[29] Anunciação P. C., Ribeiro R. C. L., Pereira M. Q., Comunian M. (2014). Different measurements of waist circumference and sagittal abdominal diameter and their relationship with cardiometabolic risk factors in elderly men. *Journal of Human Nutrition and Dietetics*.

[30] Ricci N. A., Pessoa G. S., Ferriolli E., Dias R. C., Perracini M. R. (2014). Frailty and cardiovascular risk in community-dwelling elderly: a population-based study. *Clinical Interventions in Aging*.

[31] Moretto M. C., Alves RMA R. M. A., Neri A. L., Guariento M. H. (2012). Relação entre estado nutricional e fragilidade em idosos brasileiros. *Revista da Sociedade Brasileira de Clínica Médica*.

[32] Closs V. E., Rosemberg L. S., Ettrich B. G., Gomes I., Schwanke C. H. (2015). Medidas antropométricas em idosos assistidos na atenção básica e sua associação com gênero, idade e síndrome da fragilidade: dados do EMI-SUS. *Scientia Medica*.

[33] Collard R. M., Boter H., Schoevers R. A., Oude Voshaar R. C. (2012). Prevalence of frailty in community-dwelling older persons: a systematic review. *Journal of the American Geriatrics Society*.

