# Supplementary material for: Anthropometric Measures and Frailty Prediction in the Elderly: An Easy-to-Use Tool
Source: Curr Gerontol Geriatr Res. 2017 Nov 20;2017:8703503. doi: 10.1155/2017/8703503 (PMC5735592; doi:10.1155/2017/8703503)
Supplement: Supplementary file 1 — EXCEL spreadsheet based on the parameters of neural network model and built to predict the outcome. [file 8703503.f1.docx]

**Supplementary appendix S1.** Excel spreadsheet-neural network

Input data, and results.

| **Age** |  |
| --- | --- |
| **Weight** |  |
| **Waist Circumference** |  |
| **Bicipital Skinfold** |  |
| **Sagittal Abdominal Diameter** |  |
|  |  |
|  |  |
|  |  |
| **Youden Index criteria** | **Robust** |
| **Sensitivity criteria** | **Robust** |
| **Specificity criteria** | **Robust** |

Models

|  | Age | Weight | WC | BS | SAD |  | Fried | ADJUST R |
| --- | --- | --- | --- | --- | --- | --- | --- | --- |
| 1 | 67 | 75,4 | 97 | 6 | 23,5 |  | 0 | 0,496763036 |
| 2 | 75 | 32,4 | 57,5 | 2 | 14,5 |  | 1 | 0,885873555 |
| 4 | 72 | 55 | NA | 8 | 21,8 |  | 1 | NA |
| 5 | 62 | 75,9 | 101,7 | 11 | 25,2 |  | 0 | 0,844958198 |
| 6 | 60 | 70,1 | 95 | 10 | 23,5 |  | 1 | 0,060061936 |
| 7 | 65 | 108,6 | 127 | 15 | 33,2 |  | 1 | 0,999553366 |
| 8 | 83 | 67,2 | 100 | 10,5 | 25,3 |  | 1 | 0,885864817 |
| 9 | 80 | 52,2 | 94 | 7 | 21,3 |  | 0 | 0,885873121 |
| 10 | 77 | 70 | 97,5 | 13,5 | 24,4 |  | 0 | 0,885807726 |
| 11 | 62 | 79 | 97,4 | 7 | 22,3 |  | 1 | 0,384719038 |
| 12 | 61 | 69,6 | 96,5 | 17 | 25,3 |  | 1 | 0,974757928 |
| 13 | 60 | 80,9 | 108 | 27 | 27,3 |  | 1 | 0,999967317 |
| 14 | 68 | 49,4 | 69,5 | 6 | 17,6 |  | 0 | 0,885870326 |
| 15 | 62 | 74,9 | 92,8 | 6 | 22,5 |  | 0 | 0,363382887 |
| 16 | 61 | 70,6 | 86 | 5,5 | 22,5 |  | 0 | 0,300815945 |
| 17 | 64 | 61 | 85,5 | 3 | 20,6 |  | 0 | 0,523632689 |
| 18 | 69 | 69,5 | 96,5 | 5 | 23,5 |  | 1 | 0,524002553 |
| 20 | 78 | 56,9 | 90 | 9 | 24 |  | 0 | 0,885848923 |
|  | 84 | 73,6 | NA | NA | NA |  |  | NA |
|  | 64 | 74,3 | 97 | 4 | 26,6 |  |  | 0,495335175 |
|  | 62 | 88,6 | 104 | 7 | 25,5 |  |  | 0,496505699 |
|  | 61 | 73,9 | 92,5 | 5 | 21,8 |  |  | 0,38646157 |
|  | 68 | 62,2 | 93,5 | 4 | 23 |  |  | 0,90693632 |
|  | 67 | 66,8 | 100,5 | 19 | 26,9 |  |  | 0,971276062 |
|  | 64 | 65,5 | 96,5 | 16 | 23,3 |  |  | 0,922361167 |
|  | 66 | 62 | 97,5 | 7 | 21,5 |  |  | 0,933240551 |
|  | 70 | 55 | 89,5 | 7 | 24,1 |  |  | 0,88289749 |
|  | 72 | 64,6 | 78 | 9,5 | 20,6 |  |  | 0,885861163 |
|  | 67 | 55,5 | 84,2 | 6 | 20,6 |  |  | 0,885589199 |
|  | 60 | 89 | 121 | 12 | 31,5 |  |  | 0,999553127 |
|  | 61 | 71,7 | 109 | 10,5 | 27,6 |  |  | 0,997577178 |
|  | 61 | 85,5 | 101 | 13 | 25,6 |  |  | 0,529570171 |
|  | 69 | 76,5 | 102,5 | 7 | NA |  |  | NA |
|  | 67 | 55,2 | 92 | 3 | 21,6 |  |  | 0,346540499 |
|  | 63 | 60 | 86,5 | 5 | 21,4 |  |  | 0,515206923 |
|  | 63 | 44,5 | 72,5 | 4,5 | 18 |  |  | 0,885777018 |
|  | 61 | 77,6 | 103 | 14 | 25,6 |  |  | 0,606661826 |
|  | 78 | 56,2 | 88,2 | 5 | 18,4 |  |  | 0,885871239 |
|  | 78 | 61 | 95 | 11,5 | 22,3 |  |  | 0,885870343 |
|  | 70 | 56,7 | 87,5 | 5 | 22,5 |  |  | 0,885295124 |
|  | 81 | 72,4 | 96,5 | 12 | 26 |  |  | 0,885820206 |
|  | 62 | 89,3 | 111 | 23 | 31,5 |  |  | 0,999556371 |
|  | 67 | 111,9 | 127 | 8 | 34,5 |  |  | 0,999553002 |
|  | 62 | 88 | 112 | 18 | 28,8 |  |  | 0,999587132 |
|  | 65 | 76,9 | 106 | 19 | 28 |  |  | 0,601001838 |
|  | 65 | 70 | 83 | 8 | 21,6 |  |  | 0,386076996 |
|  | 62 | 72,4 | 88 | 5 | 21,3 |  |  | 0,491494623 |
|  | 70 | 64,9 | 93 | 10 | 24,8 |  |  | 0,879367893 |
|  | 72 | 63,7 | 102 | 5 | 26,7 |  |  | 0,993075002 |
|  | 62 | 71,4 | 103 | 9 | 23 |  |  | 0,29827034 |
|  | 63 | 44,5 | 70,5 | 3 | 16,6 |  |  | 0,885851884 |
|  | 65 | 99,3 | 116,5 | 10 | 31,9 |  |  | 0,999554903 |
|  | 64 | 58,2 | 81 | 9 | 19,5 |  |  | 0,863992938 |
|  | 69 | 85,6 | 107 | 8 | 28,5 |  |  | 0,686692258 |
|  | 62 | 86,1 | 104,5 | 17 | 30,1 |  |  | 0,997319921 |
|  | 70 | 98,1 | 116 | 13 | 31,3 |  |  | 0,239316362 |
|  | 76 | 69,2 | 94 | 5 | 22,9 |  |  | 0,387711245 |
|  | 61 | 64,2 | 94 | 4 | 23,5 |  |  | 0,478340506 |
|  | 73 | 60,8 | 89 | 9 | 22,5 |  |  | 0,885795767 |
|  | 72 | 50,1 | 76 | 4 | 18,2 |  |  | 0,885872568 |
|  | 77 | 47,2 | 80 | 6 | 19,7 |  |  | 0,885873281 |
|  | 60 | 83 | 104 | 11 | 24,5 |  |  | 0,686213176 |
|  | 75 | 79,3 | 101,5 | 20 | 25,5 |  |  | 0,883156494 |
|  | 60 | 46,2 | 74 | 4 | 18,8 |  |  | 0,884377261 |
|  | 67 | 64,1 | 93 | 13 | 21,4 |  |  | 0,348745066 |
|  | 66 | 58,6 | 79 | 3,5 | 21 |  |  | 0,384555118 |
|  | 64 | 85,4 | 105 | 23,5 | 26,8 |  |  | 0,917593634 |
|  | 60 | 75,8 | 88,5 | 6 | 21,3 |  |  | 0,407483992 |
|  | 63 | 96,4 | 117 | 14 | 28 |  |  | 0,998715927 |
|  | 68 | 82,5 | 106,5 | 15 | 26,4 |  |  | 0,258935796 |
|  | 100 | 53,7 | 89,5 | 12 | 23,1 |  |  | 0,885873555 |
|  | 64 | 78 | 93,5 | 6 | 22,5 |  |  | 0,470575154 |
|  | 61 | 91,1 | 114,5 | 20 | 28,8 |  |  | 0,99958082 |
|  | 65 | 60,1 | 93,5 | 5 | 23,5 |  |  | 0,909051177 |
|  | 82 | 75,7 | 100 | 7 | 25,5 |  |  | 0,386806925 |
|  | 60 | 93,8 | 118 | 22 | 27,2 |  |  | 0,999650912 |
|  | 70 | 85,9 | NA | NA | NA |  |  | NA |
|  | 65 | 96,4 | 120 | 25 | 34 |  |  | 0,999553786 |
|  | 60 | 95 | 109,5 | 15 | 29,2 |  |  | 0,999558962 |
|  | 76 | 95,2 | 114 | 21 | 31,8 |  |  | 0,992572268 |
|  | 78 | 64,6 | 92 | 7 | 22,8 |  |  | 0,885864041 |
|  | 60 | 69,2 | 100,5 | 14 | 23,6 |  |  | 0,218130922 |
|  | 61 | 60,2 | 91 | 10 | 23,5 |  |  | 0,750316513 |
|  | 71 | 58 | 86 | 12 | 21,8 |  |  | 0,885805754 |
|  | 60 | 78,4 | 99,5 | 5 | 23,8 |  |  | 0,797295133 |
|  | 61 | 66,4 | 87 | 6 | 20 |  |  | 0,522904099 |
|  | 64 | 77,3 | 109 | 22 | 27,8 |  |  | 0,94351407 |
|  | 73 | 69,3 | 86 | 13 | 21,5 |  |  | 0,885855653 |
|  | 62 | 83,6 | 112,5 | 8 | 29 |  |  | 0,999565109 |
|  | 65 | 64,5 | 101 | 11 | 26,3 |  |  | 0,996129462 |
|  | 70 | 70 | 93 | 6 | 21,9 |  |  | 0,384265547 |
|  | 64 | 79,3 | 102 | 7 | 25,8 |  |  | 0,822593174 |
|  | 66 | 81,1 | 101 | 9 | 26,8 |  |  | 0,865319366 |
|  | 69 | 79,1 | 102,5 | 22 | 26 |  |  | 0,921406808 |
|  | 61 | 66,3 | 98 | 12 | 24,8 |  |  | 0,90731627 |
|  | 66 | 69,5 | 107 | 12 | 24,5 |  |  | 0,898038658 |
|  | 75 | 75,1 | NA | 8 | NA |  |  | NA |
|  | 63 | 46,2 | 76,5 | 3 | 17,5 |  |  | 0,885441421 |
|  | 73 | 58,9 | 97 | 9 | NA |  |  | NA |
|  | 71 | 52,6 | 95,5 | 17 | 25,4 |  |  | 0,883140194 |
|  | 62 | 72,8 | 102 | 5 | 29 |  |  | 0,208043922 |
|  | 64 | 79,4 | 103,5 | 8 | 26,5 |  |  | 0,587460501 |
|  | 60 | 71 | 103 | 10 | 30,2 |  |  | 0,999555061 |
|  | 63 | 56,5 | 77 | 5 | 18,5 |  |  | 0,865742342 |
|  | 62 | 83,5 | 108 | 8 | NA |  |  | NA |
|  | 63 | 96,8 | 110,5 | 7 | 27,4 |  |  | 0,24115777 |
|  | 71 | 98,2 | NA | 17 | NA |  |  | NA |
|  | 75 | 70 | 106,5 | 13 | 24,2 |  |  | 0,349369567 |
|  | 62 | 67,2 | 95,3 | 4 | 22,4 |  |  | 0,365271947 |
|  | 76 | 75,9 | 103 | 16 | 26,5 |  |  | 0,884597719 |
|  | 70 | 82,8 | 107,3 | 14 | NA |  |  | NA |
|  | 69 | 79 | 97 | 17 | 25,2 |  |  | 0,507400834 |
|  | 67 | 85,9 | 106,5 | 16 | 27,4 |  |  | 0,935343835 |
|  | 66 | 71,4 | 101 | 14 | 23,5 |  |  | 0,92687064 |
|  | 76 | 48,7 | 84,5 | 6 | 20,9 |  |  | 0,885871368 |
|  | 70 | 64,9 | 97 | 14 | 24,3 |  |  | 0,881842596 |
|  | 64 | 58,5 | 91 | 8 | 22,1 |  |  | 0,330909599 |
|  | 62 | 56,1 | 88,4 | 9 | 21 |  |  | 0,336548113 |
|  | 69 | 70,5 | 95,5 | 12 | 23 |  |  | 0,37579355 |
|  | 68 | 101,7 | 129,5 | 13 | 31,7 |  |  | 0,999566602 |
|  | 80 | 66,5 | 112 | 14 | NA |  |  | NA |
|  | 75 | 46,8 | 71,5 | 9 | 18,5 |  |  | 0,885873555 |
|  | 80 | 69,9 | 107 | 13 | NA |  |  | NA |
|  | 71 | 69,2 | 94,4 | 5 | 24,4 |  |  | 0,937200058 |
|  | 70 | 53,6 | 87 | 13 | 20,5 |  |  | 0,885851491 |
|  | 65 | 64,9 | 95,8 | 6 | 23,6 |  |  | 0,433759745 |
|  | 63 | 91 | 106,5 | 11 | 26,6 |  |  | 0,404857889 |
|  | 77 | 89,3 | 110,5 | 21 | 27,9 |  |  | 0,528533065 |
|  | 77 | 55,3 | 88 | 5,5 | 23 |  |  | 0,885855035 |
|  | 65 | 69,3 | 107 | 5,5 | 25,5 |  |  | 0,960960288 |
|  | 75 | 70,5 | 92,5 | 8 | 22,2 |  |  | 0,884434096 |
|  | 78 | 68,7 | 104,5 | 8,5 | 27,4 |  |  | 0,883524348 |
|  | 79 | 53,1 | 86 | 8 | 19,9 |  |  | 0,885873555 |
|  | 82 | 81,3 | 101 | 5 | 24,2 |  |  | 0,044477852 |
|  | 70 | 74,9 | 99,5 | 10 | 24,9 |  |  | 0,511574261 |
|  | 65 | 62,2 | 91 | 12 | 22,8 |  |  | 0,341715787 |
|  | 66 | 71 | 100 | 7 | 26,1 |  |  | 0,970315115 |
|  | 63 | 49,1 | 80 | 5 | 19,5 |  |  | 0,885200149 |
|  | 68 | 83,3 | 113 | 16 | 29,1 |  |  | 0,536518715 |
|  | 63 | 73,9 | 101 | 19 | 29,3 |  |  | 0,815986182 |
|  | 60 | 52,3 | 72 | 3 | 17,8 |  |  | 0,883339973 |
|  | 69 | 55,5 | 83 | 8,5 | 18,5 |  |  | 0,884905281 |
|  | 70 | 73,5 | 92 | 6 | 21,8 |  |  | 0,535786619 |
|  | 60 | 55,9 | 83 | 4,5 | 20,5 |  |  | 0,858175728 |
|  | 65 | 81,6 | 93 | 10 | 22,3 |  |  | 0,515588066 |
|  | 84 | 50 | 85 | 7 | 22,8 |  |  | 0,885873555 |
|  | 70 | 79,8 | 107 | 17 | 28 |  |  | 0,19540502 |
|  | 62 | 73,1 | 108,5 | 11 | NA |  |  | NA |
|  | 64 | 69 | 94,5 | 6,5 | 23 |  |  | 0,444840659 |
|  | 61 | 72,3 | 102,5 | 7,5 | 25,5 |  |  | 0,465533403 |
|  | 66 | 69,4 | 95 | 7 | 24,6 |  |  | 0,365430974 |
|  | 79 | 96,5 | 115,5 | 12 | NA |  |  | NA |
|  | 60 | 51,9 | 84,5 | 7 | 21 |  |  | 0,309444372 |
|  | 71 | 55,4 | 82 | 7 | 20 |  |  | 0,885860818 |
|  | 64 | 71,5 | 94 | 5,5 | 24,1 |  |  | 0,233701138 |
|  | 72 | 94,4 | 121 | 11 | 30,4 |  |  | 0,475009149 |
|  | 85 | 61,4 | 88 | 4,5 | 24 |  |  | 0,885872726 |
|  | 65 | 70 | 97,3 | 11 | 23,6 |  |  | 0,466513736 |
|  | 77 | 47,6 | 85 | 4 | 20,7 |  |  | 0,88587234 |
|  | 62 | 67,7 | 94 | 15 | 25 |  |  | 0,921074687 |
|  | 66 | 60,1 | 92 | 5 | 21,5 |  |  | 0,369752258 |
|  | 77 | 52,3 | NA | 4,5 | NA |  |  | NA |
|  | 84 | 52 | NA | 5 | NA |  |  | NA |
|  | 68 | 67,3 | 76 | 10,5 | 19,2 |  |  | 0,38683721 |
|  | 78 | 80 | 103,5 | 13 | 26,6 |  |  | 0,385202821 |
|  | 65 | 92,9 | 111,5 | 17,5 | 27,8 |  |  | 0,47751342 |
|  | 78 | 66,8 | 101,5 | 9 | NA |  |  | NA |
|  | 71 | 66,6 | 92,5 | 6,5 | 20,6 |  |  | 0,349737104 |
|  | 71 | 80 | 104,5 | 8 | 28,5 |  |  | 0,961579337 |
|  | 67 | 59,5 | 84,5 | 7 | 20,4 |  |  | 0,882417892 |
|  | 64 | 74,7 | 98 | 12 | 22,5 |  |  | 0,501093411 |
|  | 69 | 77,1 | 98,5 | 4,5 | 21,7 |  |  | 0,534609188 |
|  | 61 | 82,9 | 96,5 | 8,5 | 24,4 |  |  | 0,855160615 |
|  | 63 | 78,4 | 101,5 | 24 | 24,5 |  |  | 0,872868169 |
|  | 62 | 45,5 | 78,5 | 3,5 | 19 |  |  | 0,885174026 |
|  | 73 | 61,5 | 90 | 6 | 21,2 |  |  | 0,885796968 |
|  | 70 | 97,8 | 111,5 | 11 | 28,8 |  |  | 0,745798898 |
|  | 73 | 53,2 | 90 | 7 | 22 |  |  | 0,885826583 |
|  | 73 | 82 | 106 | 5,5 | 26,5 |  |  | 0,473074793 |
|  | 70 | 78,4 | 97 | 6 | 27,3 |  |  | 0,999179964 |
|  | 71 | 51,1 | 80 | 3 | NA |  |  | NA |
|  | 76 | 94,2 | 106 | 17 | 27,6 |  |  | 0,520832615 |
|  | 63 | 80,7 | NA | 7 | NA |  |  | NA |
|  | 62 | 59,7 | 84 | 5 | 23,6 |  |  | 0,791602507 |
|  | 73 | 69,3 | 107,5 | 11 | 29,4 |  |  | 0,999921821 |
|  | 72 | 52,7 | 81 | 3 | 20,3 |  |  | 0,885858896 |
|  | 65 | 99,5 | 121 | 26 | 34,3 |  |  | 0,999553525 |
|  | 72 | 52,6 | 85,4 | 5 | 20,6 |  |  | 0,885855302 |
|  | 79 | 58,4 | 83,8 | 8 | 21,6 |  |  | 0,885872667 |
|  | 80 | 57,1 | 93,2 | 5 | 24,5 |  |  | 0,885849817 |
|  | 67 | 70,1 | 104,5 | 9 | 26,2 |  |  | 0,673144584 |
|  | 82 | 46,4 | 75,2 | 5 | 18,2 |  |  | 0,885873555 |
|  | 65 | 65,4 | 90,3 | 23 | 25,6 |  |  | 0,815049494 |
|  | 72 | 68,1 | 106,5 | 14 | 26,5 |  |  | 0,925677653 |
|  | 74 | 93,7 | 120,5 | 11,5 | 33,4 |  |  | 0,228510965 |
|  | 61 | 46 | 76 | 3 | 18,2 |  |  | 0,885361142 |
|  | 73 | 93,2 | 121 | 9 | 30,5 |  |  | 0,548905117 |
|  | 65 | 66,7 | 97,5 | 14,5 | 22,2 |  |  | 0,932140265 |
|  | 66 | 66,6 | 95 | 14 | 23 |  |  | 0,917221858 |
|  | 83 | 47,4 | 84 | 10 | 21 |  |  | 0,885873555 |
|  | 89 | 45,4 | 77 | 4 | 19,4 |  |  | 0,885873555 |
|  | 60 | 78,4 | 101,5 | 16 | 26 |  |  | 0,392458588 |
|  | 85 | 59,1 | 97,5 | 14 | 24,8 |  |  | 0,885872697 |
|  | 69 | 48,6 | 84,5 | 6 | 20 |  |  | 0,885833764 |
|  | 66 | 66,9 | 89,5 | 5 | 21,7 |  |  | 0,529685427 |
|  | 73 | 60 | 91,5 | 5,5 | 20,5 |  |  | 0,350760057 |
|  | 62 | 60 | 84 | 3,5 | 21,4 |  |  | 0,495056642 |
|  | 77 | 88,8 | 107,5 | 16 | 29,4 |  |  | 0,92612829 |
|  | 83 | 80 | 106,5 | 11,5 | 26,7 |  |  | 0,885811123 |
|  | 70 | 67,6 | 84 | 5 | 19,5 |  |  | 0,041946796 |
|  | 85 | 50,5 | 88 | 10 | 20,4 |  |  | 0,885873555 |
|  | 65 | 83,7 | 102,5 | 6,5 | 26 |  |  | 0,864328158 |
|  | 95 | 52,3 | 83 | 6 | 22,7 |  |  | 0,885873555 |
|  | 66 | 70,3 | 89 | 3,5 | 21,5 |  |  | 0,529696193 |
|  | 71 | 67,7 | 86,5 | 6,5 | 23,2 |  |  | 0,385057384 |
|  | 71 | 79,2 | 102,1 | 8 | 28 |  |  | 0,991111709 |
|  | 69 | 78,8 | 103,5 | 6,5 | 24,6 |  |  | 0,48296879 |
|  | 73 | 45,9 | 68 | 5,5 | 17 |  |  | 0,885873555 |
|  | 71 | 77,4 | 95,5 | 4 | 24,7 |  |  | 0,515387946 |
|  | 64 | 49,2 | 77 | 3,5 | 20,4 |  |  | 0,884674096 |
|  | 71 | 57,6 | 94,5 | 12 | 22,4 |  |  | 0,885544932 |
|  | 69 | 51 | 78 | 4 | 19 |  |  | 0,885859225 |
|  | 75 | 74 | NA | 14 | NA |  |  | NA |
|  | 64 | 55,2 | 82 | 4 | 18,8 |  |  | 0,349323604 |
|  | 61 | 74,3 | 101,5 | 10 | 22,8 |  |  | 0,24965554 |
|  | 64 | 74,7 | 83 | 3 | 20,5 |  |  | 0,52972732 |
|  | 70 | 72,8 | 96,5 | 8 | 25,2 |  |  | 0,532443391 |
|  | 71 | 77,5 | 99 | 9 | 24 |  |  | 0,530725495 |
|  | 63 | 52,4 | 76,5 | 13 | 19 |  |  | 0,885717149 |
|  | 71 | 88,3 | 115 | 7,5 | 28,4 |  |  | 0,926363201 |
|  | 72 | 86,1 | 112 | 22 | 30,5 |  |  | 0,902807176 |
|  | 64 | 79,4 | 101,5 | 5 | 29 |  |  | 0,217344272 |
|  | 78 | 42 | 73 | 4,5 | 17,2 |  |  | 0,885873555 |
|  | 60 | 63,2 | 87 | 5 | 22,8 |  |  | 0,142351909 |
|  | 64 | 54,9 | 94,5 | 12 | 23,9 |  |  | 0,530931406 |
|  | 66 | 67,4 | 96,5 | 11,5 | 23,6 |  |  | 0,925086407 |
|  | 76 | 98,9 | 116,5 | 9 | 30,8 |  |  | 0,918404656 |
|  | 70 | 60,2 | 91 | 4 | 23 |  |  | 0,884530291 |
|  | 66 | 76,4 | 108 | 21 | 28,3 |  |  | 0,971762553 |
|  | 73 | 63,3 | 101,5 | 16 | 26,6 |  |  | 0,880132776 |
|  | 60 | 108,9 | 119 | 13 | NA |  |  | NA |
|  | 64 | 61,6 | 92 | 12 | 23 |  |  | 0,313231795 |
|  | 63 | 81,3 | 103,5 | 20 | 26,3 |  |  | 0,873243351 |
|  | 66 | 81,5 | 115,5 | 18 | 27,4 |  |  | 0,859409168 |
|  | 67 | 60 | 87,5 | 5,5 | 24,1 |  |  | 0,451982102 |
|  | 79 | 61 | 97 | 17,5 | 22,3 |  |  | 0,885872566 |
|  | 66 | 81,4 | 94,5 | 6 | 22,8 |  |  | 0,502755375 |
|  | 62 | 57,5 | 82,3 | 8 | 18 |  |  | 0,349406589 |
|  | 83 | 44,5 | NA | 2 | NA |  |  | NA |
|  | 61 | 76,3 | 107 | 11 | 24,3 |  |  | 0,911206627 |
|  | 63 | 68 | 99,5 | 15 | 24,5 |  |  | 0,785219628 |
|  | 61 | 101,3 | 117 | 9 | 29 |  |  | 0,999558036 |
|  | 68 | 73,8 | 101,5 | 5 | 25,5 |  |  | 0,304539023 |
|  | 64 | 75,6 | 105,5 | 10,5 | 27,5 |  |  | 0,372636028 |
|  | 70 | 84,4 | 103,5 | 12 | 27,6 |  |  | 0,211637318 |
|  | 63 | 122,1 | 149 | 25,5 | NA |  |  | NA |
|  | 61 | 58,6 | 84 | 7,5 | 20,6 |  |  | 0,333254931 |
|  | 61 | 76,8 | 97 | 5,5 | 21,9 |  |  | 0,354512128 |
|  | 69 | 94,1 | 109 | 18 | 28,1 |  |  | 0,962455962 |
|  | 61 | 85,9 | 110,9 | 10 | 29,5 |  |  | 0,999558064 |
|  | 68 | 64,3 | 97 | 6,5 | 22,5 |  |  | 0,933085113 |
|  | 67 | 64,8 | 89,3 | 12 | 22,4 |  |  | 0,87954719 |
|  | 77 | 60,3 | 91,8 | 12 | 22,2 |  |  | 0,885869182 |
|  | 71 | 68,7 | 102,9 | 18 | 26 |  |  | 0,761478468 |
|  | 67 | 76,6 | 102 | 9 | 25 |  |  | 0,366982515 |
|  | 61 | 75 | 103 | 17 | 24,7 |  |  | 0,245295639 |
|  | 64 | 77,1 | 108 | 7 | 26,9 |  |  | 0,416357699 |
|  | 61 | 93,4 | 102 | 8 | 25,2 |  |  | 0,45131084 |
|  | 64 | 80,7 | 101,5 | 15 | 26,4 |  |  | 0,86348166 |
|  | 81 | 68,5 | NA | 4 | NA |  |  | NA |
|  | 64 | 79,1 | 105 | 7 | 25,5 |  |  | 0,888658328 |
|  | 76 | 60,7 | 99,5 | 6 | 23,5 |  |  | 0,881351558 |
|  | 73 | 73,2 | 87,5 | 16 | 24,1 |  |  | 0,885532666 |
|  | 79 | 58 | 88 | 8 | NA |  |  | NA |
|  | 86 | 62,9 | 101,5 | 16 | NA |  |  | NA |
|  | 66 | 63,8 | 87,5 | 4,5 | 20 |  |  | 0,04175843 |
|  | 70 | 49,5 | 77 | 7 | 19,9 |  |  | 0,885857856 |
|  | 80 | 77,7 | 96 | 4 | 23 |  |  | 0,321686186 |
|  | 83 | 74,8 | 106 | 12 | 24,6 |  |  | 0,831632768 |
|  | 68 | 58,2 | 90 | 12 | 20,6 |  |  | 0,350067328 |
|  | 70 | 73,3 | 97 | 13 | 25,8 |  |  | 0,932800681 |
|  | 65 | 79,5 | 105 | 21 | 26,4 |  |  | 0,090579664 |
|  | 70 | 51,4 | 86,5 | 12 | 21 |  |  | 0,885832247 |
|  | 61 | 53,1 | 80,1 | 6 | 19 |  |  | 0,347372723 |
|  | 84 | 76,2 | 103 | 10 | 25,5 |  |  | 0,885864932 |
|  | 66 | 62,5 | 92 | 11,5 | 22,9 |  |  | 0,342805542 |
|  | 71 | 57,4 | 87,5 | 12 | 22,6 |  |  | 0,885699583 |
|  | 61 | 66,1 | 103 | 12 | 25,5 |  |  | 0,959375981 |
|  | 76 | 47 | 71,5 | 3 | 17,4 |  |  | 0,885873555 |
|  | 62 | 66,7 | 95 | 10 | 22,6 |  |  | 0,421685038 |
|  | 66 | 49 | 86 | 10 | 19,8 |  |  | 0,885395323 |
|  | 72 | 38,2 | NA | NA | NA |  |  | NA |
|  | 80 | 66,2 | 91 | 6 | 21,5 |  |  | 0,885872776 |
|  | 63 | 72,5 | NA | NA | NA |  |  | NA |
|  | 66 | 54,5 | 84,5 | 3,5 | 20 |  |  | 0,418602706 |
|  | 69 | 73,1 | 108,5 | 9 | 24,8 |  |  | 0,486126826 |
|  | 63 | 70 | 95,6 | 14 | 26,5 |  |  | 0,945840341 |
|  | 75 | 61 | 93 | 7 | 23,5 |  |  | 0,885764511 |
|  | 86 | 54,4 | 94,5 | 15 | NA |  |  | NA |
|  | 75 | 75,1 | 95 | 9 | 24,2 |  |  | 0,38622699 |
|  | 67 | 80,3 | 103,8 | 22 | 25,5 |  |  | 0,909399674 |
|  | 67 | 55,4 | 87 | 16 | 22,5 |  |  | 0,88497233 |
|  | 84 | 65,5 | 87 | 5,5 | 21 |  |  | 0,885873555 |
|  | 69 | 64,1 | 92 | 17 | 23,5 |  |  | 0,884751625 |
|  | 83 | 70 | 95,5 | 8 | 24,8 |  |  | 0,88586732 |
|  | 60 | 71,8 | 96 | 8 | 24,4 |  |  | 0,754356579 |
|  | 65 | 51,1 | 76 | 5 | 18 |  |  | 0,885836572 |
|  | 67 | 80,2 | 117,2 | 15 | 28,2 |  |  | 0,64868335 |
|  | 73 | 84,6 | 107,6 | 17 | 29,2 |  |  | 0,431281831 |
|  | 68 | 87,5 | 108 | 28 | 27,5 |  |  | 0,271750645 |
|  | 73 | 81,7 | 113 | 18 | 29,1 |  |  | 0,244027415 |
|  | 65 | 85,7 | 113,5 | 16 | 29,3 |  |  | 0,252088724 |
|  | 78 | 78 | 106,5 | 14 | 28 |  |  | 0,882570927 |
|  | 73 | 57,7 | 98,5 | 4 | 22,7 |  |  | 0,349399612 |
|  | 71 | 79,8 | 106 | 17 | 26,6 |  |  | 0,477194407 |
|  | 65 | 57,6 | 91,5 | 6 | 21,3 |  |  | 0,34445381 |
|  | 69 | 63,6 | 94 | 15 | 24,3 |  |  | 0,882219118 |
|  | 64 | 100 | 110 | 9 | 28,5 |  |  | 0,224415399 |
|  | 66 | 73,5 | 106,7 | 19 | 26,4 |  |  | 0,704445732 |
|  | 86 | 36,9 | NA | 3 | NA |  |  | NA |
|  | 73 | 83,9 | 104 | 21 | 26,2 |  |  | 0,528353207 |
|  | 60 | 69 | 101 | 21 | 26 |  |  | 0,948416379 |
|  | 62 | 96,5 | 104 | 7,5 | 28 |  |  | 0,215454724 |
|  | 67 | 74,7 | 101,5 | 5 | 26,1 |  |  | 0,979960735 |
|  | 79 | 69,9 | 101,7 | 16 | 24,4 |  |  | 0,885855835 |
|  | 63 | 71,3 | 94,4 | 8 | 23,5 |  |  | 0,309869283 |
|  | 63 | 80,1 | 100,7 | 17 | 25 |  |  | 0,108388487 |
|  | 72 | 37 | 65 | 4 | 15,3 |  |  | 0,885873555 |
|  | 66 | 54,3 | 85 | 3 | 21 |  |  | 0,884674609 |
|  | 71 | 82,7 | 109 | 6 | 26,3 |  |  | 0,386206932 |
|  | 71 | 44,2 | 76,8 | 8 | 19,5 |  |  | 0,885869085 |
|  | 64 | 70,2 | 95,5 | 12 | 23 |  |  | 0,482820625 |
|  | 72 | 68 | 92,5 | 13 | 24 |  |  | 0,885243822 |
|  | 61 | 71,6 | 93,5 | 20 | 21,9 |  |  | 0,925562178 |
|  | 71 | 63,2 | 94,5 | 4 | 23,2 |  |  | 0,361718149 |
|  | 67 | 84,7 | 105,5 | 11 | 27,8 |  |  | 0,707833053 |
|  | 76 | 58,2 | 93,5 | 5,5 | 24,2 |  |  | 0,885717579 |
|  | 84 | 73,2 | 97,2 | 7 | 25 |  |  | 0,885867997 |
|  | 76 | 65,7 | 98,5 | 9 | 23,4 |  |  | 0,881157529 |
|  | 70 | 54,8 | 86,5 | 8 | 21,3 |  |  | 0,885775878 |
|  | 70 | 62,5 | 93,8 | 4,5 | 23,7 |  |  | 0,876303368 |
|  | 62 | 72,8 | 94,8 | 21 | 25,5 |  |  | 0,718626814 |
|  | 64 | 46,4 | 76,4 | 7 | 19 |  |  | 0,885723264 |
|  | 80 | 78,9 | 107 | 10 | 25,8 |  |  | 0,042695394 |
|  | 75 | 48,8 | 73,5 | 4 | 17,9 |  |  | 0,885873555 |
|  | 66 | 116,8 | 141 | 20 | NA |  |  | NA |
|  | 71 | 61,2 | 96,5 | 23,5 | 24,1 |  |  | 0,8855062 |
|  | 70 | 61,9 | 94 | 18 | NA |  |  | NA |
|  | 68 | 72 | 98 | 22 | 27 |  |  | 0,78085896 |
|  | 64 | 49,5 | 81 | 4 | 19,8 |  |  | 0,885250598 |
|  | 61 | 66,5 | 108 | 10 | 25,6 |  |  | 0,928015711 |
|  | 65 | 61,7 | 95 | 16 | 24,8 |  |  | 0,824820949 |
|  | 62 | 66,2 | 96 | 10 | 24,1 |  |  | 0,127465638 |
|  | 74 | 79,8 | NA | 10 | NA |  |  | NA |
|  | 70 | 52 | 88 | 12 | 22 |  |  | 0,885736455 |
|  | 60 | 65,5 | 93 | 5 | 21,9 |  |  | 0,307407686 |
|  | 68 | 70,1 | 102,5 | 11 | 25,8 |  |  | 0,746384569 |
|  | 69 | 67 | 96 | 12 | 25,4 |  |  | 0,864687463 |
|  | 67 | 68,8 | 95 | 15 | 23,5 |  |  | 0,339160003 |
|  | 67 | 79,3 | 100,5 | 13 | 25,8 |  |  | 0,280118101 |
|  | 70 | 68,1 | 94 | 5 | 21,3 |  |  | 0,215268969 |
|  | 62 | 78,6 | 99 | 10 | 24,9 |  |  | 0,895357567 |
|  | 78 | 73 | 95 | 12 | 22,5 |  |  | 0,885866397 |
|  | 78 | 60 | 88,5 | 11 | 21,9 |  |  | 0,885871736 |
|  | 70 | 63,8 | 88,5 | 7 | 21,8 |  |  | 0,885548237 |
|  | 66 | 75,7 | 97 | 16 | 24,5 |  |  | 0,463308401 |
|  | 65 | 66,3 | 95 | 14 | 21,8 |  |  | 0,933001886 |
|  | 88 | 36,6 | 73 | 7 | 17,2 |  |  | 0,885873555 |
|  | 65 | 56,8 | 87 | 9 | 20 |  |  | 0,349059729 |
|  | 75 | 71,7 | 91 | 13 | 24,3 |  |  | 0,885701018 |
|  | 70 | 82,7 | 97 | 6 | 24,4 |  |  | 0,512039327 |
|  | 60 | 58,9 | 85 | 8 | 19,6 |  |  | 0,931930998 |
|  | 62 | 42,5 | 63 | 4 | 15,6 |  |  | 0,885864801 |
|  | 67 | 95 | 113,2 | 8 | 29,9 |  |  | 0,224555751 |
|  | 64 | 70,2 | 94 | 17 | 25,6 |  |  | 0,944962827 |
|  | 61 | 73,2 | 105,5 | 12,5 | 25,3 |  |  | 0,682714714 |
|  | 62 | 65,1 | 91 | 15 | 23,4 |  |  | 0,888024593 |
|  | 66 | 60,1 | 87 | 10 | 20,4 |  |  | 0,349204708 |
|  | 63 | 68,7 | 111 | 15 | NA |  |  | NA |
|  | 75 | 74,2 | 111,5 | 12 | 29 |  |  | 0,883200701 |
|  | 67 | 83,9 | 112,5 | 10 | 26,9 |  |  | 0,91231862 |
|  | 61 | 85,5 | 104 | 8 | 24,7 |  |  | 0,668503916 |
|  | 63 | 67,9 | 94 | 9,5 | 25,9 |  |  | 0,868556168 |
|  | 60 | 79,9 | 94,6 | 16 | 26,4 |  |  | 0,334697734 |
|  | 69 | 72,4 | 96,5 | 20 | 24,6 |  |  | 0,873972128 |
|  | 69 | 58,4 | 87,3 | 6 | 20,5 |  |  | 0,754542768 |
|  | 61 | 67,9 | 102 | 17 | 26,8 |  |  | 0,885919227 |
|  | 62 | 104,5 | 116,5 | 15 | 31,7 |  |  | 0,999553588 |
|  | 64 | 75,5 | 97 | 7 | 23 |  |  | 0,431621537 |
|  | 66 | 52,2 | 89 | 8,5 | 21,1 |  |  | 0,708524287 |
|  | 69 | 74,8 | 101,9 | 12 | 22,5 |  |  | 0,543980345 |
|  | 78 | 89,4 | 113 | 22 | 30 |  |  | 0,924357111 |
|  | 62 | 60,4 | 92,3 | 11 | 23,7 |  |  | 0,824972438 |
|  | 64 | 44 | 66,7 | 3 | 17,7 |  |  | 0,885833549 |
|  | 62 | 73,7 | 98,5 | 4 | 23,9 |  |  | 0,665914274 |
|  | 63 | 73,2 | 99 | 6 | 25,7 |  |  | 0,713452389 |
|  | 60 | 94,9 | 116 | 20 | 30,4 |  |  | 0,999555171 |
|  | 60 | 77,9 | 89,5 | 6,5 | 22,6 |  |  | 0,145143709 |
|  | 61 | 61,5 | 84 | 11 | 19,5 |  |  | 0,346389292 |
|  | 61 | 78,1 | 99,7 | 17 | 25 |  |  | 0,90909523 |
|  | 62 | 90,8 | 104 | 7 | 25,6 |  |  | 0,45290261 |
|  | 63 | 61,6 | 87 | 4 | 21,1 |  |  | 0,518910175 |
|  | 66 | 64,3 | 86 | 16 | 20,4 |  |  | 0,401068047 |
|  | 76 | 65 | 90 | 8 | 24,7 |  |  | 0,885645923 |
|  | 66 | 56,9 | 92,5 | 6,5 | 22,9 |  |  | 0,334144336 |
|  | 66 | 86,7 | 112,8 | 5 | 28,4 |  |  | 0,267064876 |
|  | 63 | 71,7 | 102,5 | 6 | 25,8 |  |  | 0,660328028 |
|  | 64 | 51,9 | 81,5 | 4 | 20 |  |  | 0,885017069 |
|  | 67 | 66,7 | 97 | 6 | 25,1 |  |  | 0,349109387 |
|  | 61 | 57,1 | 89 | 6 | 22,4 |  |  | 0,877325232 |
|  | 62 | 54,3 | 82,5 | 5,5 | 19,9 |  |  | 0,345401489 |
|  | 62 | 77,2 | 96 | 6,5 | 25,5 |  |  | 0,611739242 |
|  | 69 | 74,2 | 111 | 10 | 26,7 |  |  | 0,210651029 |
|  | 68 | 64,2 | 94,5 | 4,5 | 20,6 |  |  | 0,934249089 |
|  | 103 | NA | NA | NA | NA |  |  | NA |
|  | 76 | 54,4 | 88 | 4,5 | 22,8 |  |  | 0,885845307 |
|  | 75 | 67,2 | 92 | 6,5 | 26,4 |  |  | 0,378349935 |
|  | 70 | NA | NA | NA | NA |  |  | NA |
|  | 71 | 60,5 | 91,5 | 10 | 22,5 |  |  | 0,885648105 |
|  | 67 | 59 | 91,5 | 5 | 21 |  |  | 0,348406611 |
|  | 95 | NA | NA | NA | NA |  |  | NA |
|  | 63 | 80 | 98 | 7,5 | 26,5 |  |  | 0,440292912 |
|  | 87 | NA | NA | NA | NA |  |  | NA |
|  | 70 | 55,9 | 88 | 4 | 22 |  |  | 0,885513715 |
|  | 71 | 67,6 | 94,5 | 16 | 24,4 |  |  | 0,884565 |
|  | 67 | 67 | 95,5 | 10 | 23,3 |  |  | 0,930222786 |
|  | 70 | 71,4 | 88 | 3 | 19,6 |  |  | 0,041894731 |
|  | 60 | 113,3 | 125 | 22 | 33 |  |  | 0,999552928 |
|  | 79 | 83 | 109,8 | 6 | 28,4 |  |  | 0,51590389 |
|  | 63 | 97,7 | 115,5 | 23 | 31,3 |  |  | 0,999558698 |
|  | 65 | 75,7 | 110 | 10 | 27 |  |  | 0,636692009 |
|  | 60 | 64,4 | 99 | 8 | 24,4 |  |  | 0,784976038 |
|  | 63 | 89,4 | 126 | 12 | 31 |  |  | 0,999555231 |
|  | 60 | 68,1 | NA | 5 | NA |  |  | NA |
|  | 85 | 51,5 | NA | 7 | NA |  |  | NA |
|  | 67 | 81,2 | 108 | 13 | 28,9 |  |  | 0,40552791 |
|  | 63 | 49,6 | NA | 7 | NA |  |  | NA |
|  | 62 | 73,7 | 100,5 | 17 | 25,2 |  |  | 0,91602065 |
|  | 63 | 70,5 | 93 | 7,5 | 23 |  |  | 0,396907314 |
|  | 67 | 86,2 | 104 | 14 | 28,5 |  |  | 0,548671793 |
|  | 74 | 57 | 88 | 6 | NA |  |  | NA |
|  | 85 | 61,6 | 88 | 13 | 23,5 |  |  | 0,885873555 |
|  | 64 | 68,6 | 95,5 | 7 | 24,5 |  |  | 0,181270846 |
|  | 73 | 114 | 133,5 | 12,5 | 36,2 |  |  | 0,999553709 |
|  | 63 | 62,6 | 83 | 15 | 21,5 |  |  | 0,882606822 |
|  | 63 | 67,2 | 95 | 5 | 25 |  |  | 0,931909468 |
|  | 65 | 77,5 | 104 | 14 | 26 |  |  | 0,881542224 |
|  | 79 | 62,3 | 84,5 | 6 | 20,5 |  |  | 0,885873211 |
|  | 68 | 78,6 | 105 | 11 | 26,8 |  |  | 0,66920603 |
|  | 66 | 84,9 | 95 | 6 | 24,5 |  |  | 0,310159844 |
|  | 64 | 62,7 | 89,5 | 13 | 23 |  |  | 0,839881479 |
|  | 65 | 85,6 | 107,5 | 4 | 26 |  |  | 0,746931867 |
|  | 62 | 71,5 | 90 | 9 | 22,8 |  |  | 0,380420339 |
|  | 78 | 62,5 | 81 | 7 | 21,5 |  |  | 0,885871956 |
|  | 68 | 61,2 | 87 | 9 | 21 |  |  | 0,88028501 |
|  | 65 | 67,5 | 94 | 4 | 24,2 |  |  | 0,304934775 |
|  | 61 | 65,4 | 91,5 | 11 | NA |  |  | NA |
|  | 61 | 67,4 | 99,5 | 11 | 24 |  |  | 0,072934081 |
|  | 61 | 70,4 | 99 | 11 | 25,2 |  |  | 0,733680797 |
|  | 63 | 55,9 | 77,5 | 3 | 17,9 |  |  | 0,349500398 |
|  | 61 | 67,2 | 95 | 4 | 26,9 |  |  | 0,245953988 |
|  | 69 | 70,7 | 93 | 6 | 23,7 |  |  | 0,523244875 |
|  | 70 | 80,5 | 104 | 8,5 | 25,3 |  |  | 0,478990273 |
|  | 73 | 60,7 | 89 | 3,5 | NA |  |  | NA |
|  | 63 | 82,9 | 110 | 14 | 28,5 |  |  | 0,248242172 |
|  | 80 | 71,1 | 101,5 | 10 | 24 |  |  | 0,885551958 |
|  | 76 | 68,5 | 105,5 | 8,5 | 27,9 |  |  | 0,872396253 |
|  | 71 | 95 | 112,5 | 12 | 30,3 |  |  | 0,433027727 |
|  | 72 | 80 | 101 | 6 | 26,8 |  |  | 0,413148127 |
|  | 60 | 52,3 | 85 | 14 | 20,7 |  |  | 0,358702489 |
|  | 70 | 64,1 | 105 | 12 | 24,9 |  |  | 0,929875566 |
|  | 68 | 58,2 | 91,5 | 4 | 21,8 |  |  | 0,347599717 |
|  | 69 | 74,5 | 94,5 | 5 | 23,1 |  |  | 0,528119719 |
|  | 62 | 64 | 83,5 | 19 | 20,5 |  |  | 0,491506989 |
|  | 62 | 89,6 | 118,5 | 10 | 28,8 |  |  | 0,999568675 |
|  | 68 | 75,7 | 93,8 | 13 | 25,8 |  |  | 0,907155345 |
|  | 66 | 69,7 | 96 | 9 | 24,1 |  |  | 0,451835521 |
|  | 81 | 52,4 | NA | 9 | NA |  |  | NA |
|  | 78 | 57,1 | 78 | 5 | 19,5 |  |  | 0,885873555 |
|  | 74 | 78,7 | 97 | 6 | 24,8 |  |  | 0,535500752 |
|  | 68 | 71,3 | 90 | 5 | 23,2 |  |  | 0,523828587 |
|  | 69 | 75,3 | 98 | 15 | 23,4 |  |  | 0,557798196 |
|  | 81 | 42,3 | 63 | 2 | 16,5 |  |  | 0,885873555 |
|  | 63 | 54,5 | 87,5 | 6 | 23,8 |  |  | 0,743425055 |
|  | 79 | 57,2 | 87,5 | 5 | 21,6 |  |  | 0,885872253 |
|  | 75 | 81,4 | 101,6 | 22 | 26,2 |  |  | 0,884974444 |
|  | 66 | 90 | 113 | 18 | 26,8 |  |  | 0,930030235 |
|  | 70 | 73,3 | 108 | 10 | 24,5 |  |  | 0,929340073 |
|  | 71 | 82 | 99,5 | 10 | 28,8 |  |  | 0,996878035 |
|  | 62 | 92,5 | 122,1 | 20 | 29,9 |  |  | 0,999564484 |
|  | 63 | 106,5 | 127 | 12 | 33,2 |  |  | 0,999552975 |
|  | 66 | 58,7 | 86 | 3 | 21 |  |  | 0,345870565 |
|  | 78 | 63,7 | 85 | 12,5 | 22,7 |  |  | 0,885869647 |
|  | 74 | 57,3 | 82,5 | 3 | 19,9 |  |  | 0,885870128 |
|  | 62 | 57,5 | 96 | 5 | 26,4 |  |  | 0,899410525 |
|  | 75 | 65,2 | 94,5 | 6 | 23,6 |  |  | 0,885718707 |
|  | 79 | 63,1 | 86 | 6 | 21,4 |  |  | 0,885872533 |
|  | 67 | 65,9 | 100,5 | 3,5 | 25,7 |  |  | 0,781811575 |
|  | 61 | 76,1 | 100,2 | 14 | 25,7 |  |  | 0,599443922 |
|  | 72 | 57,2 | 92 | 8 | 23,2 |  |  | 0,885536584 |
|  | 69 | 57,6 | 86 | 15 | 20,8 |  |  | 0,88582652 |
|  | 68 | 80,9 | 101 | 21 | 24,7 |  |  | 0,515054102 |
|  | 61 | 61,8 | 88,5 | 15 | 22,8 |  |  | 0,295456652 |
|  | 68 | 58,4 | 97,5 | 4,5 | NA |  |  | NA |
|  | 62 | 90,4 | 102 | 7 | 27,5 |  |  | 0,22927849 |
|  | 69 | 60,6 | NA | 4 | NA |  |  | NA |
|  | 62 | 72,8 | 98 | 6 | 22,8 |  |  | 0,294274757 |
|  | 63 | 86,6 | 105 | 7 | 26,3 |  |  | 0,412710281 |
|  | 78 | 54 | 83 | 4 | 19,8 |  |  | 0,885873284 |
|  | 68 | 62,5 | 99,5 | 9 | 24 |  |  | 0,928468443 |
|  | 68 | 50,3 | 88 | 5 | 23,2 |  |  | 0,882338362 |
|  | 62 | 77,1 | 100 | 7 | 24,3 |  |  | 0,934594015 |
|  | 61 | 64,6 | 81 | 13 | 21,9 |  |  | 0,342716396 |
|  | 64 | 68,5 | 104,6 | 11 | 26 |  |  | 0,914702681 |
|  | 67 | 70,6 | 94 | 15 | 23,6 |  |  | 0,861672253 |
|  | 70 | 69,7 | 101 | 11 | 29,8 |  |  | 0,963107237 |
|  | 63 | 59,4 | 84,7 | 10 | 20,9 |  |  | 0,361241552 |
|  | 85 | 85,9 | 105 | 24 | 28,8 |  |  | 0,885811954 |
|  | 89 | 77,3 | NA | 17 | NA |  |  | NA |
|  | 74 | 87,9 | NA | 12 | NA |  |  | NA |
|  | 65 | 88,2 | 104 | 7 | 24,4 |  |  | 0,193133233 |
|  | 76 | 59,4 | 96,5 | 14 | 24,5 |  |  | 0,885778526 |
|  | 70 | 76,5 | NA | 5 | NA |  |  | NA |
|  | 62 | 99,3 | 114,5 | 7 | 27,4 |  |  | 0,528463801 |
|  | 88 | 59,3 | 88,5 | 3,5 | 21,5 |  |  | 0,885873555 |
|  | 67 | 69,9 | 100 | 13 | 25,5 |  |  | 0,880551895 |
|  | 62 | 55,6 | 80,1 | 10 | 19,6 |  |  | 0,883666371 |
|  | 88 | 46,5 | 76 | 4 | 19,8 |  |  | 0,885873555 |
|  | 74 | 87,8 | 106,5 | 10 | 28,2 |  |  | 0,369048789 |
|  | 60 | 91,2 | NA | 16 | NA |  |  | NA |
|  | 61 | 86,2 | 103,5 | 12 | 26,7 |  |  | 0,276184208 |
|  | 61 | 66,9 | 103 | 10 | 25,4 |  |  | 0,616354771 |
|  | 66 | 87,3 | 107,5 | 13 | 27 |  |  | 0,849192609 |
|  | 67 | 63 | 96,6 | 11 | 24,6 |  |  | 0,544675553 |
|  | 61 | 75,1 | 107 | 16 | 24,1 |  |  | 0,077080877 |
|  | 68 | 75 | 93 | 11 | 23,5 |  |  | 0,524604549 |
|  | 66 | 83,3 | 118,5 | 28 | 30 |  |  | 0,999974533 |
|  | 69 | 78,7 | 104,5 | 24 | 25,5 |  |  | 0,928538892 |
|  | 73 | 44 | 66,5 | 4,5 | 19 |  |  | 0,885872602 |
|  | 63 | 78,8 | 112 | 12 | 27,7 |  |  | 0,278195699 |
|  | 62 | 79 | 113 | 23 | 27,4 |  |  | 0,999715965 |
|  | 61 | 69,4 | 98,6 | 7 | 25,7 |  |  | 0,431808108 |
|  | 68 | 62,4 | 97,6 | 5 | 22,2 |  |  | 0,933340891 |
|  | 65 | 60,1 | 84,5 | 8 | 22,2 |  |  | 0,880847489 |
|  | 70 | 61,7 | 98,6 | 11 | 26,4 |  |  | 0,844058699 |
|  | 67 | 80 | 105 | 16 | 25 |  |  | 0,440774096 |
|  | 67 | 69 | 105,5 | 7,5 | 26 |  |  | 0,347707308 |
|  | 78 | 78,6 | 103 | 16 | 26,3 |  |  | 0,88552572 |
|  | 62 | 75 | 98 | 15,5 | 23 |  |  | 0,409848934 |
|  | 60 | 92,8 | 124,5 | 14 | 31,8 |  |  | 0,999553058 |

| ENTRY |  | OUTPUT |  |
| --- | --- | --- | --- |
| -92,58200861 | H1 | 129,5717378 | 1 |
| -7,451461792 | H2 |  |  |
| 82,60197085 | H3 |  |  |
| 14,37480464 | H4 |  |  |
| -155,0295366 | H5 |  |  |
| 48,23331099 | H6 |  |  |
|  |  |  |  |
| 6,19647E-41 |  | -3,360587096 | 0,033550182 |
| 0,000580255 |  |  |  |
| 1 |  |  |  |
| 0,999999428 |  |  |  |
| 4,69383E-68 |  |  |  |
| 1 |  |  |  |
|  |  |  |  |
| PAGE 143 |  |  |  |

Synaptic Weights

|  | x |  |  |  |  | Entry | Output |  |  |
| --- | --- | --- | --- | --- | --- | --- | --- | --- | --- |
| 1 | -3,812351943 | B-H1 |  | -3,812351943 | B-H1 | 1 | -3,81235194 |  |  |
| 2 | 7,073850844 | I1-H1 |  | 7,073850844 | I1-H1 | 0 | 0 |  |  |
| 3 | -3,574930243 | I2-H1 |  | -3,574930243 | I2-H1 | 0 | 0 |  |  |
| 4 | -9,563168806 | I3-H1 |  | -9,563168806 | I3-H1 | 0 | 0 |  |  |
| 5 | 1,22423912 | I4-H1 |  | 1,22423912 | I4-H1 | 0 | 0 |  |  |
| 6 | 26,68570283 | I5-H1 |  | 26,68570283 | I5-H1 | 0 | 0 |  |  |
| 7 | -56,80782806 | B-H2 |  | -56,80782806 | B-H2 | 1 | -56,8078281 |  |  |
| 8 | 0,243839009 | I1-H2 |  | 0,243839009 | I1-H2 | 0 | 0 |  |  |
| 9 | -0,362558595 | I2-H2 |  | -0,362558595 | I2-H2 | 0 | 0 |  |  |
| 10 | 0,327764413 | I3-H2 |  | 0,327764413 | I3-H2 | 0 | 0 |  |  |
| 11 | 0,834137664 | I4-H2 |  | 0,834137664 | I4-H2 | 0 | 0 |  |  |
| 12 | 1,002472198 | I5-H2 |  | 1,002472198 | I5-H2 | 0 | 0 |  |  |
| 13 | 1,492883007 | B-H3 |  | 1,492883007 | B-H3 | 1 | 1,492883007 |  |  |
| 14 | 18,79433067 | I1-H3 |  | 18,79433067 | I1-H3 | 0 | 0 |  |  |
| 15 | -14,5923942 | I2-H3 |  | -14,5923942 | I2-H3 | 0 | 0 |  |  |
| 16 | -0,661774558 | I3-H3 |  | -0,661774558 | I3-H3 | 0 | 0 |  |  |
| 17 | 6,506533329 | I4-H3 |  | 6,506533329 | I4-H3 | 0 | 0 |  |  |
| 18 | -2,242196268 | I5-H3 |  | -2,242196268 | I5-H3 | 0 | 0 |  |  |
| 19 | 0,000845964 | B-H4 |  | 0,000845964 | B-H4 | 1 | 0,000845964 |  |  |
| 20 | 0,048609703 | I1-H4 |  | 0,048609703 | I1-H4 | 0 | 0 |  |  |
| 21 | 0,055789374 | I2-H4 |  | 0,055789374 | I2-H4 | 0 | 0 |  |  |
| 22 | 0,067078223 | I3-H4 |  | 0,067078223 | I3-H4 | 0 | 0 |  |  |
| 23 | -0,001438408 | I4-H4 |  | -0,001438408 | I4-H4 | 0 | 0 |  |  |
| 24 | 0,017558835 | I5-H4 |  | 0,017558835 | I5-H4 | 0 | 0 |  |  |
| 25 | -1,030057215 | B-H5 |  | -1,030057215 | B-H5 | 1 | -1,03005722 |  |  |
| 26 | 0,341215444 | I1-H5 |  | 0,341215444 | I1-H5 | 0 | 0 |  |  |
| 27 | -9,913344084 | I2-H5 |  | -9,913344084 | I2-H5 | 0 | 0 |  |  |
| 28 | 2,848293157 | I3-H5 |  | 2,848293157 | I3-H5 | 0 | 0 |  |  |
| 29 | -15,30690158 | I4-H5 |  | -15,30690158 | I4-H5 | 0 | 0 |  |  |
| 30 | 16,43243417 | I5-H5 |  | 16,43243417 | I5-H5 | 0 | 0 |  |  |
| 31 | -2,712013354 | B-H6 |  | -2,712013354 | B-H6 | 1 | -2,71201335 |  |  |
| 32 | 7,678822159 | I1-H6 |  | 7,678822159 | I1-H6 | 0 | 0 |  |  |
| 33 | -14,41302461 | I2-H6 |  | -14,41302461 | I2-H6 | 0 | 0 |  |  |
| 34 | 6,626532493 | I3-H6 |  | 6,626532493 | I3-H6 | 0 | 0 |  |  |
| 35 | 0,808148835 | I4-H6 |  | 0,808148835 | I4-H6 | 0 | 0 |  |  |
| 36 | -1,038989355 | I5-H6 |  | -1,038989355 | I5-H6 | 0 | 0 |  |  |
| 37 | 0,102690401 | B-O |  |  |  |  |  |  |  |
| 38 | 1,254061144 | H1-O |  |  |  | Entry | Control | Entry | Output |
| 39 | 1,553190585 | H2-O |  | 0,102690401 | B-O | 1 | 1,000000 | 1,000000 | 0,102690401 |
| 40 | -3,875518218 | H3-O |  | 1,254061144 | H1-O | -3,812351943 | -3,812352 | 0,021618 | 0,027110876 |
| 41 | 0,145975288 | H4-O |  | 1,553190585 | H2-O | -56,80782806 | -56,807828 | 0,000000 | 0 |
| 42 | 2,063020194 | H5-O |  | -3,875518218 | H3-O | 1,492883007 | 1,492883 | 0,816511 | -3,164401713 |
| 43 | 3,302206677 | H6-O |  | 0,145975288 | H4-O | 0,000845964 | 0,000846 | 0,500211 | 0,073018517 |
|  |  |  |  | 2,063020194 | H5-O | -1,030057215 | -1,030057 | 0,263073 | 0,542724936 |
|  |  |  |  | 3,302206677 | H6-O | -2,712013354 | -2,712013 | 0,062268 | 0,20562242 |

|  |  |  |  |  |  | **Cutt-off point** |
| --- | --- | --- | --- | --- | --- | --- |
| Age | 0 |  | Youden Index criteria | -2,21323456 | 0,098568 | **0,7176** |
| Weight | 0 |  | Specificity criteria | -2,21323456 | 0,098568 | **0,5648** |
| Waist Circumference | 0 |  | Sensitivity criteria | -2,21323456 | 0,098568 | **0,5621** |
| Bicipital Skinfold | 0 |  |  |  |  |  |
| Sagittal Abdominal Diameter | 0 |  |  |  |  |  |
|  |  |  |  |  |  |  |
|  |  |  | Results |  |  |  |
|  |  |  | If higher than the cutt-off point |  |  | **Prefrail or Frail** |
|  |  |  | If minor than the cutt-off point |  |  | **Robust** |
